# Structural-Guided Identification of Small Molecule Inhibitor of UHRF1 Methyltransferase Activity

**DOI:** 10.3389/fgene.2022.928884

**Published:** 2022-08-03

**Authors:** Md Abdul Awal, Suza Mohammad Nur, Ali Khalaf Al Khalaf, Mohd Rehan, Aamir Ahmad, Salman Bakr I. Hosawi, Hani Choudhry, Mohammad Imran Khan

**Affiliations:** ^1^ Department of Biochemistry, Faculty of Science, King Abdulaziz University, Jeddah, Saudi Arabia; ^2^ King Fahd Medical Research Centre, King Abdulaziz University, Jeddah, Saudi Arabia; ^3^ Translational Research Institute, Hamad Medical Corporation, Doha, Qatar; ^4^ Centre of Artificial Intelligence for Precision Medicines, King Abdulaziz University, Jeddah, Saudi Arabia

**Keywords:** UHRF1, SRA domain, chicoric acid, global methylation (5 mC), molecular docking, molecular dynamics simulation

## Abstract

Ubiquitin-like containing plant homeodomain Ring Finger 1 (UHRF1) protein is recognized as a cell-cycle-regulated multidomain protein. UHRF1 importantly manifests the maintenance of DNA methylation mediated by the interaction between its SRA (SET and RING associated) domain and DNA methyltransferase-1 (DNMT1)-like epigenetic modulators. However, overexpression of UHRF1 epigenetically responds to the aberrant global methylation and promotes tumorigenesis. To date, no potential molecular inhibitor has been studied against the SRA domain. Therefore, this study focused on identifying the active natural drug-like candidates against the SRA domain. A comprehensive set of *in silico* approaches including molecular docking, molecular dynamics (MD) simulation, and toxicity analysis was performed to identify potential candidates. A dataset of 709 natural compounds was screened through molecular docking where chicoric acid and nystose have been found showing higher binding affinities to the SRA domain. The MD simulations also showed the protein ligand interaction stability of and *in silico* toxicity analysis has also showed chicoric acid as a safe and nontoxic drug. In addition, chicoric acid possessed a longer interaction time and higher LD50 of 5000 mg/kg. Moreover, the global methylation level (%5 mC) has been assessed after chicoric acid treatment was in the colorectal cancer cell line (HCT116) at different doses. The result showed that 7.5 µM chicoric acid treatment reduced methylation levels significantly. Thus, the study found chicoric acid can become a possible epidrug-like inhibitor against the SRA domain of UHRF1 protein.

## Introduction

The DNA methylation like epigenetic modification in the CpG island manifests a crucial role in mammalian genomic architecture, genomes expression, and genome stability (Chen et al., 1998; Bird and Wolffe, 1999). Moreover, diverse biological responses including tumorigenesis are associated with multiple patterns of DNA methylation (Arita et al., 2008). Among several epigenetic modulators, the DNA methyltransferase family (DNMT) is one of the key components that play with epigenetic modification. It is well established that DNMT1 acts as a canonical epigenetic ‘writer’ (Patnaik et al., 2018) in the DNA methylation mechanism (Moore et al., 2012). Additionally, DNMT1 incorporates methyl group at the fifth position of cytosine in CpG islands and synthesizes 5-methylcytosine (5 mC). Moreover, DNMT1 maintains DNA methylation during the DNA replication phase (Pradhan and Esteve, 2003). In contrast, aberrant DNA hypomethylation and hypermethylation are associated with transcriptional activation and repression of gene expression, respectively (Baylin et al., 2001; Patnaik et al., 2018).

Ubiquitin-like containing PHD Ring Finger 1 (UHRF1) is an essential partner protein of DNA methyltransferase and is also known as Np95 (Nuclear Protein 95 KDa) and ICBP90 (Inverted CCAAT box-binding Protein of 90 KDa) in mouse and human, respectively (Veland and Chen, 2017). Structurally, UHRF1 is a complex of distinct domains that include-SRA (SET and RING-associated) domain, a ubiquitin-like (UBL) domain, a plant homeodomain (PHD) domain, and a RING domain (Bostick et al., 2007; Bronner et al., 2007; Sharif et al., 2007; Zhang et al., 2011; Cheng et al., 2013). Moreover, UHRF1 belonging SRA domain recognizes the hemimethylated sequence in DNA and facilitates the DNMT1 binding, thus the maintenance of DNA methylation dynamics (Bostick et al., 2007).

The genomic abundance of DNA methylation disturbs normal cell division and complies with the pathogenic responses (Kong et al., 2019). It has been revealed that UHRF1 can regulate the DNA methylation in both normal and tumor cells by providing accompanying to the epigenetic writer- DNMT1 (Bostick et al., 2007; Sharif et al., 2007; Liao et al., 2015; Cai et al., 2017). Moreover, aberrant DNA methylation leads the alteration of the gene expression and has been considered as a fundamental regulator of tumor progression (Baylin and Jones, 2016). Moreover, global hypermethylation dictates cell proliferation in tumorigenesis through silencing tumor suppressor genes (TSGs) and its promoter (Shen and Laird, 2013; Baylin and Jones, 2016). Previous studies also revealed that UHRF1 protein is expressed during cellular propagation and can regulate the cell cycle (Bonapace et al., 2002; Patnaik, Estève and Pradhan, 2018). Moreover, another study also revealed that the expression of UHRF1 is required by the cell during for S-phase (Bonapace et al., 2002). However, G0/G1 phases may not notably require the UHRF1 (Uemura et al., 2000; Miura et al., 2001). UHRF1 protein is also found to be highly expressed in cancer cells across the cell cycle. For example, the overexpression of UHRF1 has been reported in several cancer-cell like-gastric, bladder, breast, lung, prostate, pancreatic, and colorectal cancer (Crnogorac-Jurcevic et al., 2005; Unoki et al., 2010; Jazirehi, Arle and Wenn, 2012; Kofunato et al., 2012; Li et al., 2012; Yang et al., 2012; Zhou et al., 2013).

Moreover, the study also revealed that overexpression of UHRF1 is associated with the DNA methylation-mediated silencing of tumor suppressor genes (Beck et al., 2018). A study also showed that by recruiting several repressor enzymes, such as DNA methyltransferase 1 (DNMT1), histone deacetylase 1 (HDAC1), and histone lysine methyltransferases, i.e., G9a and Suv39H1, UHRF1 mediates the gene silencing mechanism (Alhosin et al., 2016). Additionally, UHRF1 has been substantially justified for chemotherapeutic targets (Unoki, 2011).

Chemotherapeutic resistance is a bottleneck problem in modern cancer therapies. The chemo-resistance is influenced through several mechanisms such as chemo target alterations, signaling pathway diversion, and the inactivation of cell death (Holohan et al., 2013; Ahamed et al., 2022). The chemoresistance is explored either by the innate response which is raised through pre-existing factors in tumor cells or by the adaptive response due to mutated expression of molecular target and therapeutic insensitivity to the target (Longley and Johnston, 2005; Ahamed et al., 2021). Likewise, epigenetic modifications of histone, such as acetylation and methylation, generate a range of drug insensitivity (Housman et al., 2014). For instance, aberrant methylation of the MDR1 promoter is related to structural variations of chromatin and transcriptional repression (Baker and El-Osta, 2003). Similarly, long-term use of 5-Azacytidine (AZA) like DNMTi acquires resistance (Singh and Yu, 2018). However, small inhibitors targeting UHRF1, promote the sensitivity of therapeutics and elevate cancer inhibition (Abdullah et al., 2021).

Hemimethylated CpG sites are target sequences for the maintenance of DNA methylation and become completely methylated by UHRF1. Among other domains, The SET and RING-associated (SRA) domain of UHRF1 identifies the 5-methylcytosine (5 mC) in hemimethylated CpG sequences (Arita et al., 2008; Avvakumov et al., 2008; Hashimoto et al., 2008; Qian et al., 2008; Frauer et al., 2011). The SRA domain recognizes the presence of methylated cytosine and regulates the recruitment of DNMT1 (Arita et al., 2008; Hashimoto et al., 2008; Qian et al., 2008; Frauer et al., 2011; Bronner, Krifa and Mousli, 2013). Indeed, the SRA domain shows direct interaction with DNMT1 and catalyzes the methylation function (Berkyurek et al., 2014). It has been shown that the activity of DNMT1 has been accelerated by 1.9-fold due to the SRA domain and 5-fold because of UHRF1 (Bashtrykov et al., 2014). Therefore, the SRA domain has been identified as a potential target for inhibiting the aberrant global DNA methylation (Bashtrykov et al., 2014). Natural small molecules would not only a promising therapeutics against the SRA domain but also can possess the least side effects as anti-cancer drugs (Das and Singal, 2004).

Beyond the traditional drug discovery strategy, *in-silico* drug design has gained the attraction of concern due to time and cost management (Lin, Li and Lin, 2020). In contrast, natural chemical extraction and characterization for anticancer drug development are frequently time-consuming and include several inevitable barriers (Fang et al., 2018). Computer-aided drug design (CADD) solves this constraint by making it simple to screen, identify, and describe novel drug candidates within a short amount of time (Ahammad et al., 2019). CADD-mediated therapeutic development against lung and prostate cancer, for example, has been previously reported (Cui et al., 2020). Molecular docking and molecular dynamics (MD) simulation-based approaches are used in the CADD study to find viable therapies for various diseases (Lu et al., 2018; Shukla et al., 2021). Molecular docking analysis aids in the first screening of medication candidates for favorable binding capacity to drug-like ligands to the intended target (Kitchen et al., 2004). Similarly, MD simulations aid in understanding the stability of protein-ligand interactions in a synthetic environment that mimics the human body’s environment (Singh and Bharadvaja, 2021). As a result, computational drug design methodologies were used in this investigation to help screen out possible therapeutic candidates against the SRA domain of the UHRF1 protein.

Previously, chicoric acid-a phenolic compound derived from various plants (Lee and Scagel, 2013) has been reported as it may useful in NASH and liver fibrosis treatment (Kim et al., 2017; Pan et al., 2020). Chicoric acid as a bioactive anticancer drug in colorectal cancer has been also reported (Tsai et al., 2012). However, as an epi-drug the role of chicoric acid was overlooked. Hence, the present study is aimed to investigate the role of chicoric acid in targeting UHRF1.

## Materials and Methods

### Preparation of PDB Structures

The crystal structure of the SRA domain of E3 ubiquitin-protein ligase UHRF1 (c) was downloaded from the Protein Data Bank [ RCSB PDB: Homepage. Available online: https://www.rcsb.org/(Accessed on 25 March 2022).], the protein is then prepared and optimized by using the “Protein preparation wizard” tool of Schrödinger suite (Schrödinger, L. Schrödinger Release 2021-4: Protein Preparation Wizard; Epik, Schrödinger, LLC, New York, NY, 2021). The hydrogen bonds were added, metal bonds were deleted, zero-order bonds were added between metals and nearby atoms, and correction of the formal charges to metals and neighboring atoms was done. Then add and optimize the missing side chain by running a prime job, then generate protonation and metal charge for states for the ligands, cofactors, and metals at 7.0 ± 2.0 pH. Finally, H-bonds of hydroxyl, Asn, Gln, and His are optimized at pH 7.0 using PROPKA (Olsson et al., 2011), removal of water molecules beyond 3 Å from HET groups and OPLS4 force field has been used for minimization.

### Data Retrieval and Ligand Preparations

The natural organic compound library was retrieved from Selleckchem (https://www.selleckchem.com) as an SDF file’ 20210416-L7600-Natural-Organic-Compound-Library.sdf’ on 25 April 2021. The sdf file contained 1,126 natural organic compounds; of these, only 774 were found to have 3D structures when searched in PubChem. The 774 compounds were further filtered based on molecular weight (<500), and finally, 709 compounds were obtained and selected for virtual screening. The selected compounds were prepared using LigPrep (Schrödinger Release 2021-4: LigPrep, Schrödinger, LLC, New York, NY, 2021), 2D structures were converted to 3D, and their tautomeric forms and ionization states were generated.

### Receptor Grid Generation and Docking

Glide (Schrödinger, L. Schrödinger Release 2021-4: Glide, Schrödinger, LLC, New York, NY, 2021) was utilized for both grid generation and ligands docking. The grid was generated using the PDB: 3BI7. The binding region was specified by picking the entry identified using the SiteMap program (Schrödinger Release 2021-4: SiteMap, Schrödinger, LLC, New York, NY, 2021). The partial charge cut-off and non-polar atoms (VdW radii scaling factor) like parameters were set as 0.25 and 0.1, respectively. Molecular docking simulation has been performed by using the “ligand docking” tool in the Schrödinger suite. The selected protocol was Extra precision (XP), the ligand sampling method was flexible, and all the other settings were kept as default.

### Molecular Dynamics Simulations

Molecular dynamics (MD) simulation has been performed by using were Schrödinger suite (Schrödinger, L. Schrödinger Release 2021-4: Desmond Molecular Dynamics System, D. E. Shaw Research, New York, NY, 2021. Maestro-Desmond Interoperability Tools, Schrödinger, New York, NY, 2021), the systems of Chicoric acid (CID: 5281764) in complex with 3BI7 and Nystose (CID: 166775) were retrieved from the results of docking and first tuned through the “System Builder” tool. The orthorhombic-shaped box and TIP3P as the solvent model has been chosen. The neutralization of the system has been done with Na^+^ ions additions. Also, the slide distances box was fixed at 10 Aº. 100 ns/trajectory has been set up for the MD calculation while constantly maintaining pressure, temperature, and the number of atoms. The pressure and temperature have been set at1.01325 bar and 300.0 K, with the OPLS4 force field.

### *In Silico* Toxicity Analysis

To investigate the toxicity of chicoric acid through *in silico* analysis we availed the ProtoxII web server (https://tox-new.charite.de/protox_II/) (65). The ProTox-II web server integrates several criteria like molecular similarity, pharmacophores, fragment propensities, and machine-learning models to predict various toxicity endpoints like-cytotoxicity, hepatotoxicity, carcinogenicity, mutagenicity, immunotoxicity, adverse outcomes pathways, etc.

### Cell Culture and Dose Determination

The HCT116 cell line was collected by Dr. Imran’s Lab, Dept. of Biochemistry, KAU, Jeddah. The cell culture was performed in Dulbecco’s modified Eagle’s medium (UFS Biotech, Riyadh, KSA) supplemented with 10% FBS and 1% penicillin (Invitrogen) and incubated at 37°C. The cells were maintained up to 80–90% confluence and checked regularly to avoid any *Mycoplasma* contamination (Shait Mohammed et al., 2021). Upon confluence, cells were trypsinized and seeded in 6-well plates and incubated overnight to ensure that cells were healthy without any contamination. The next day the seeded cells were randomly treated with chicoric acid at 2.5, 5 and 7.5 µM concentrations.

### Extraction of Genomic DNA

The genomic DNA was isolated from both control (untreated) and treated HCT116 cells line by utilizing DNAbler kit (https://havensci.com/; Lot no. DE95050). 200 µL of digestion buffer was added to each sample. 20 µL of RNase A and Proteinase K were added. Afterward, the samples were vortexed and incubated in a heat block (∼60°C). Then 200 µL of lysis buffer was added followed by vortexing and centrifugation. 99% ethanol was added to aliquots. Next, ethanol-lysis buffer mix samples were collected in a spin column (nuclease-free) for further centrifugation at 10,000 g speed. After centrifugation, the samples were washed by wash buffer by following the supplied protocol of the manufacturer. 50 µL elution buffer was added to the center of the column and incubated the column at room temperature for 2 min. Then centrifugation was done to elute the genomic DNA at a speed of 8000 g for 2 min (Khan et al., 2022).

### Global Methylation (5 mC%) Level Determination

The global methylation (5 mC) level of the targeted HCT116 cell line was determined by using MethylFlash™ global DNA methylation (5-mC) ELISA Easy Kit (Catalog no. P-1030) in both treated and untreated conditions. 200 ng of DNA from each sample was collected and used for the experimental analysis. The binding solution was added to the extracted DNA samples in wells. After that, 5 mC antibody, developer solution, and stop solution were added as per manufacturer protocol. The optical density (OD) was determined at the end at 450 nm wavelength by using BioTek ELISA microplate reader (Khan et al., 2022).

## Results

### Molecular Docking Studies

**FIGURE 1:**
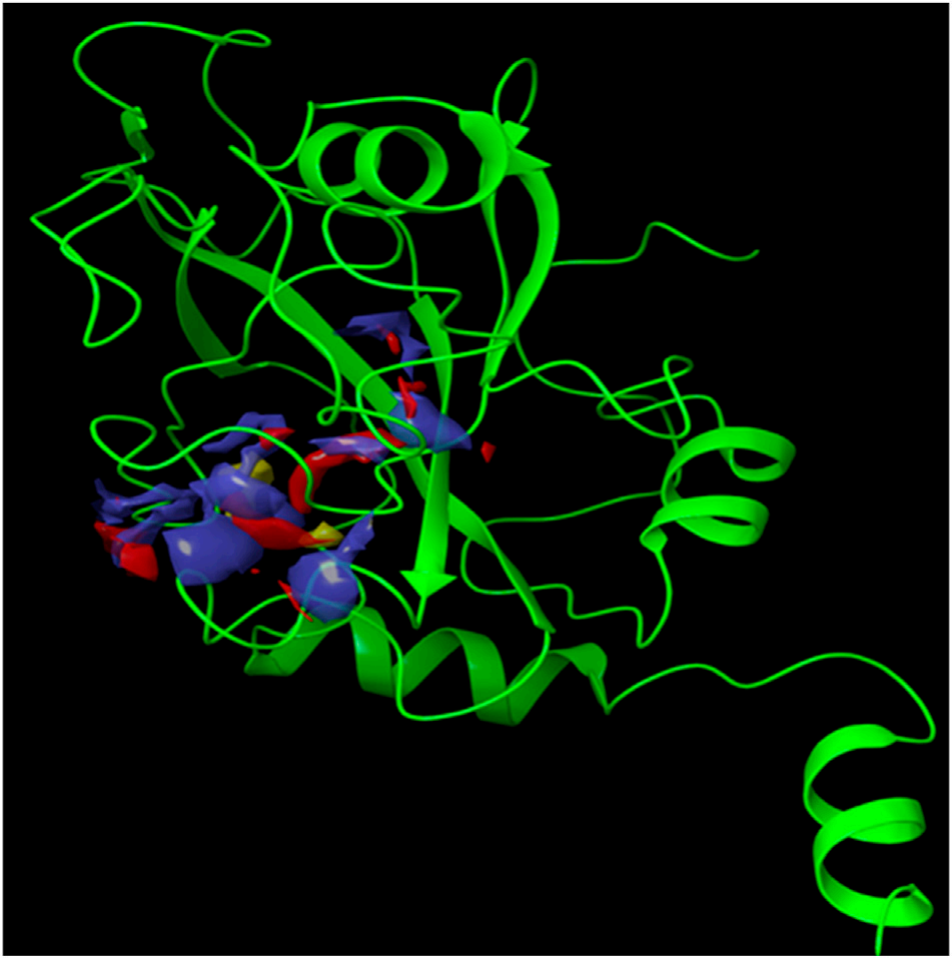
The crystal structure of the SRA domain of E3 ubiquitin-protein ligase UHRF1. SiteMap surface “red, blue and yellow colour” of the SRA domain of E3 ubiquitin-protein ligase UHRF1(PDB: 3BI7) “Green colour".

**FIGURE 2:**
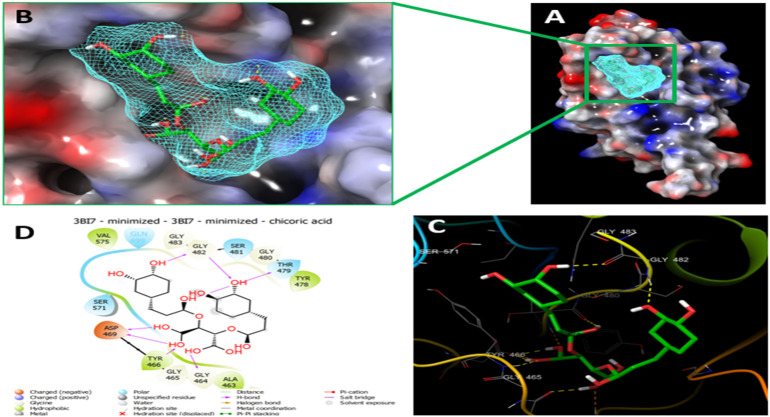
Molecular Docking of chicoric acid with UHRF1. **(A)** Molecular surface display with an electrostatic potential color scheme for UHRF1-Chicoric acid complex and the close-up view presented. **(B)** Putative binding mode of Chicoric acid in the bindin site of UHRF1(PDB: 3BI7) **(C)** Chicoric acid was displayed as green ball-and-sticks. And the amino acid residues of the are represented as grey sticks, and H-bonds are described in yellow dotted lines. **(D)** 2D depiction of the ligand-protein interactions.

Since the crystal structure of the SRA domain of E3 ubiquitin-protein ligase UHRF1(PDB: 3BI7) doesn’t contain ligands/inhibitors, and to define the Grid box, we decided to perform site mapping to identify the potential binding sites on the protein. The SiteMap program [Schrödinger Release 2021-4: SiteMap, Schrödinger, LLC, New York, NY, 2021.] detect only one site, as shown in Figure 1. Then, a grid box is generated around the detected protein’s binding site of the minimized protein by using a receptor-Grid-Generation tool in Maestro. The obtained Ligiprep file that contains 3D molecular structures of the selected compounds was docked into the protein binding site. Table 1 showed the docked ligands’ results that were selected due to their most negative docking scores, and these scores demonstrated the best-bonded ligand with relative binding affinities and conformations. Chicoric acid (CID: 5281764) and Nystose (CID: 166775) displayed the highest negative docking scores of -13.041 and -12.962 kcal/mol in complex with 3BI7, respectively. The molecular docking results showed that the chicoric acid bound well within the binding site (Figures 2A,B) with the highest negative docking scores of -13.041 and inter-acted within 3Å with 14 residues: Ala-463, Gly-464, Gly-465, Tyr-466, Asp-469, Ser-571, Val-575, Gln-499, Gly-483, Gly-482, Ser-481, Gly-480, Thr-479, Tyr-478, (Figures 2C,D). Chicoric acid form charged negative interaction with Asp-469; polar interaction with Ser-571, Gln 499, Ser 481, Thr-479; hydrophobic interaction with Ala 463, Tyr-466, Val-575, Tyr-478; Also form hydrogen bond donor interaction with Gly-464 Asp-469, Gly-482, Thr-479; and hydrogen bond acceptor interaction with Tyr-466, Gln 482.

**TABLE 1:** In silico screening/docking results of the docked ligands that were selected owing to their most negative docking scores, with SRA domain of E3 ubiquitin-protein ligase UHRF1(PDB: 3BI7).

^a^Compounds CID	Docking Score	Glide g**-**score	Glide e**-**model	XP GScore
5281764 (Chicoric acid)	-13.041	-65.303	-13.041	-13.041
166775 (Nystose)	-12.962	-60.686	-12.962	-12.962
439531	-11.978	-63.731	-11.978	-11.978
10542	-10.866	-52.983	-10.866	-10.866
5280805	-9.836	-71.019	-9.836	-9.836
5281377	-9.606	-60.709	-9.606	-9.606
4789	-10.358	-67.504	-10.358	-10.358
4789	-9.628	-61.404	-9.628	-9.628
83489	-9.374	-68.28	-9.374	-9.374
439242	-9.373	-55.967	-9.373	-9.373
6134	-9.29	-49.674	-9.29	-9.29
92817	-9.245	-59.163	-9.245	-9.245
5280704	-9.112	-65.2	-9.112	-9.112
11458	-9.013	-47.5	-9.013	-9.013
65064	-9.055	-66.577	-9.055	-9.055
160469	-8.937	-47.311	-8.937	-8.937
3085296	-8.91	-53.671	-8.91	-8.91
9476	-8.895	-49.356	-8.895	-8.895
6443665	-9.093	-58.108	-9.093	-9.093
10712	-8.67	-50.74	-8.67	-8.67
73191	-8.585	-58.472	-8.585	-8.585
5281544	-8.541	-51.528	-8.541	-8.541
5481663	-8.558	-77.148	-8.558	-8.558
65064	-9.974	-66.823	-9.974	-9.974
73395	-8.468	-68.725	-8.468	-8.468
87691	-8.338	-46.766	-8.338	-8.338
73568	-10.384	-69.056	-10.384	-10.384
125153	-8.221	-84.2	-8.221	-8.221
656569	-8.212	-44.998	-8.212	-8.212
440660	-8.207	-56.717	-8.207	-8.207
5282151	-8.145	-61.64	-8.145	-8.145
73466	-8.065	-43.983	-8.065	-8.065
736	-8.038	-36.572	-8.038	-8.038
503737	-8.015	-60.238	-8.015	-8.015

a Identifier from PubChem database of chemical molecules and their activities in biological assays.

**FIGURE 3:**
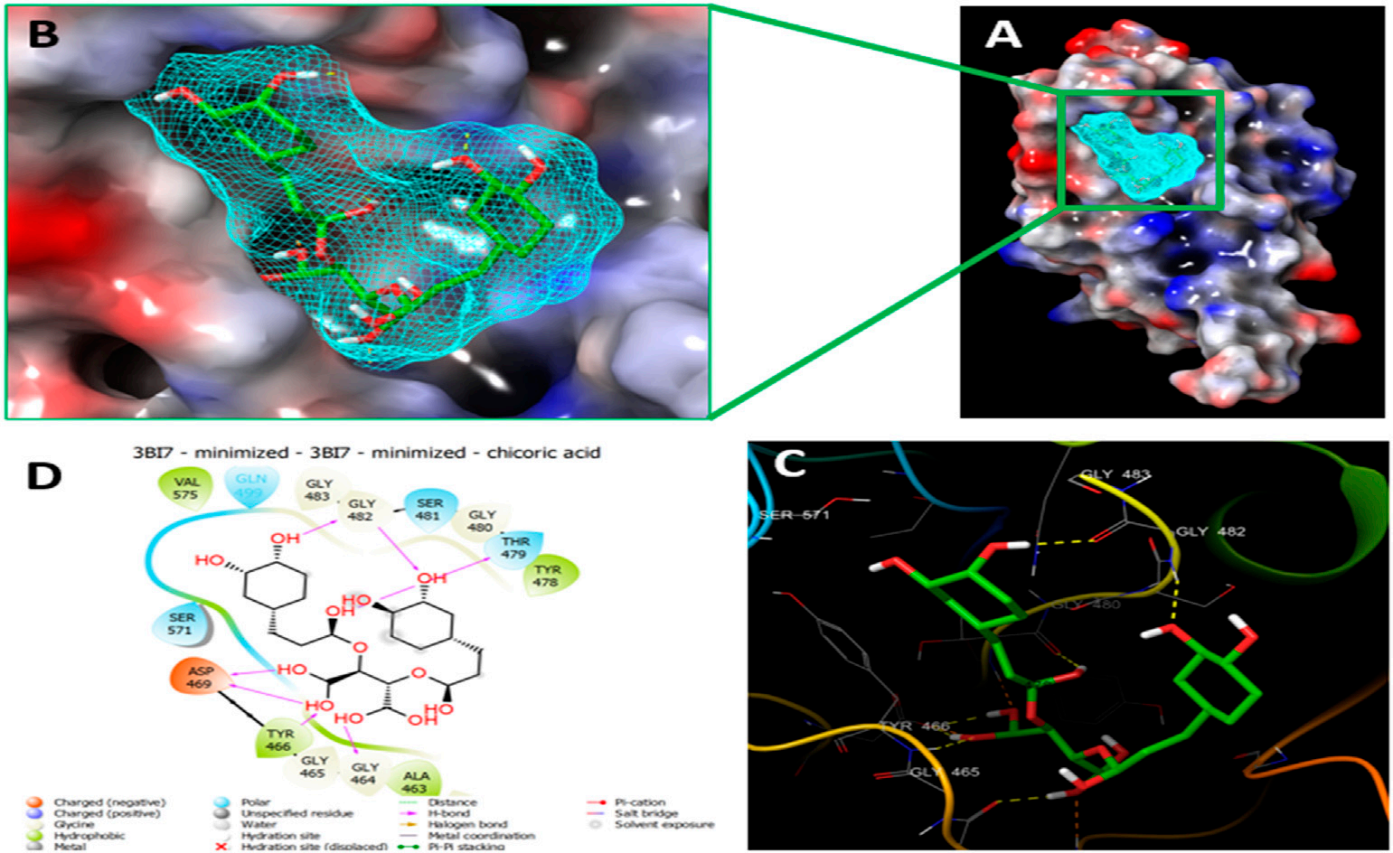
Molecular Docking of nytose with UHRF1. **(A)** Molecular surface display with an electrostatic potential color scheme for UHRF1- Nystose complex and the close-up view presented. **(B)** Putative binding mode of Nystose in the binding site of UHRF1(PDB: 3BI7). **(C)** Nystose was displayed as green ball-and-sticks. And the amino acid residues of the binding site are represented as grey sticks, and H-bonds are expressed in yellow dotted lines. **(D)** 2D depiction of the lig-and-protein interactions.

Since the molecular docking results showed that the Nystose bound well within the binding site (Figures 3A,B) with second-highest negative docking scores of -12.962 and interacted within 3Å with 16 residues: Arg-433, Gly-448, Val-446, Val-461, Leu-462, Ala-463, Gly-464, Gly-465, Tyr-466, Asp-469, Tyr-478, Thr-479, Gly-480, Ser-481, Gly-482, Gln-499 (Figures 3C,D). Nystose form charged negative interaction with Asp-469; charged positive interaction with Arg-433; polar interaction with Gln 499, Ser 481, Thr-479; hydrophobic interaction with Ala 463, Tyr-466, Val-446, Val-461, Leu-462, Tyr-478; Also form hydrogen bond donor interaction with Val-446, Val-461, Asp-469, Thr-479, Gly-480, Gly-482; and hydrogen bond acceptor interaction with Ala-463, Tyr-466.

### Molecular Dynamics Simulation

**FIGURE 4:**
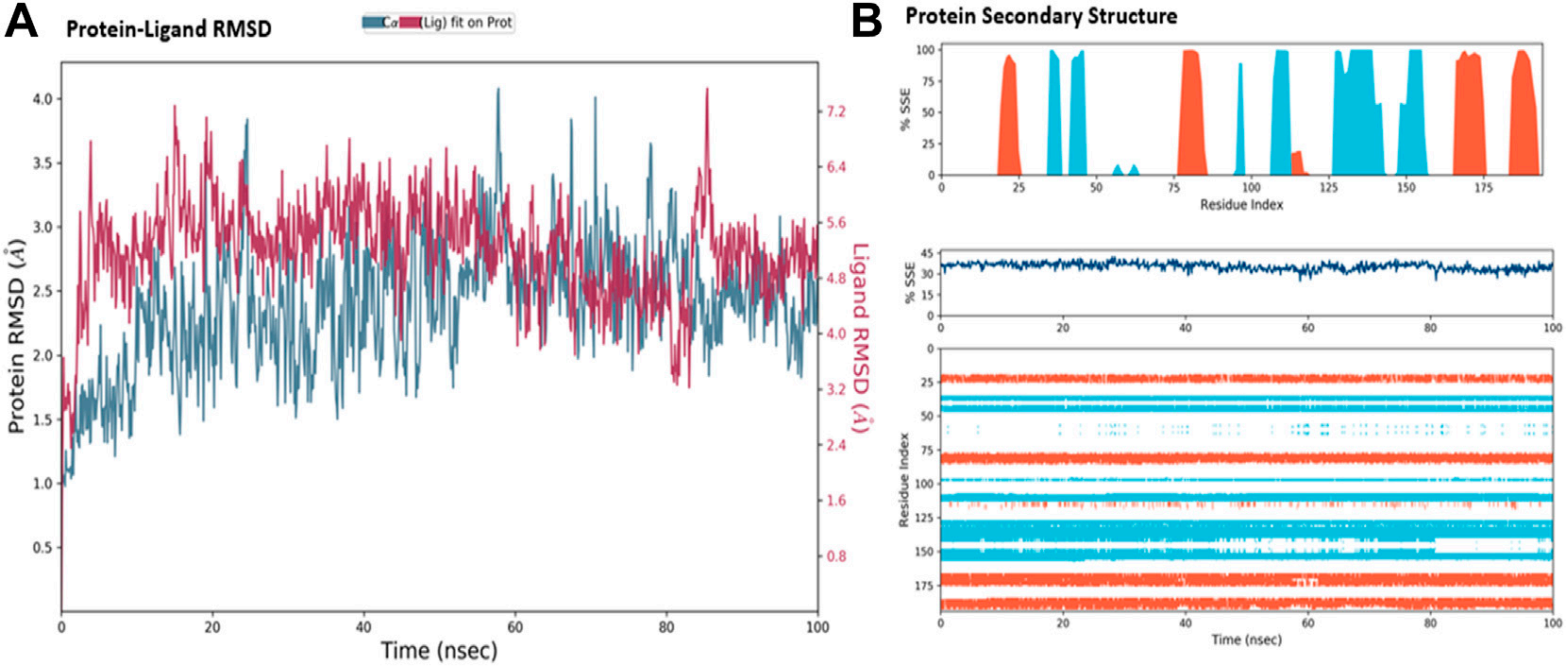
Molecular dynamics simulation analysis of chicoric acid. **(A)** The RMSD plot was obtained for compound Chicoric acid complexed with SRA domain of E3 ubiquitin-protein ligase UHRF1(PDB: 3BI7). The 100 ns simulation time reaffirmed the stability of the complex without any significant changes in the structure. **(B)** Stability of the secondary structure UHRF1 over the 100 ns of MD simulation when complexed with Chicoric acid. Protein secondary structure elements (SSE) like alpha-helices and beta-strands were monitored throughout the simulation. The top plot reported SSE distribution by residue index throughout the protein structure. The middle plot summarized the SSE composition for each trajectory frame throughout the simulation, and the plot at the bottom monitored each residue and its SSE assignment over time.

**FIGURE 5:**
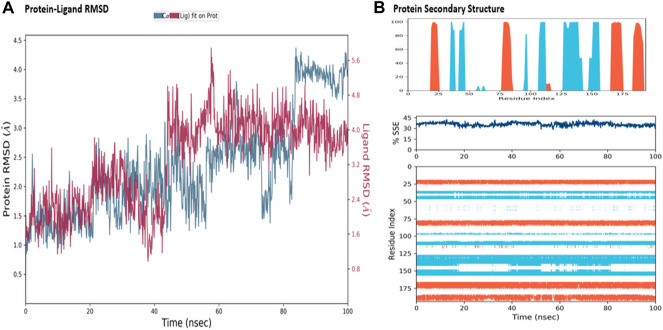
Molecular dynamics simulation analysis of nytose. **(A)** The RMSD plot was obtained for compound Nystose complexed with SRA domain of E3 ubiquitin-protein ligase UHRF1(PDB: 3BI7). The 100 ns simulation time reaffirmed the stability of the complex without any significant changes in the structure. **(B)** Stability of the secondary structure UHRF1 over the 100 ns of MD simulation when complexed with Nystose. Protein secondary structure elements (SSE) like alpha-helices and beta-strands were monitored throughout the simulation. The top plot reported SSE distribution by residue index throughout the protein structure. The middle plot summarized the SSE composition for each trajectory frame throughout the simulation, and the plot at the bottom monitored each residue and its SSE assignment over time.

The MD simulations are performed to simulate the aqueous physiological environment to assess the changes in protein conformation and binding affinity during the simulation time, compared to the original affinity and confirmation of the crystal structure (Hollingsworth and Dror, 2018). Therefore, the MD study was performed using Desmond software [Schrödinger Release 2021-4: Desmond Molecular Dynamics System, D. E. Shaw Research, New York, NY, 2021.] to evaluate the binding affinity and stability of the protein-ligand complexes at pH 7.0 ± 0.2 over 100 ns. Only the two top-scoring compounds in the docking study, i.e., Chicoric acid (CID: 5281764) and Nystose (CID: 166775), were analyzed by MD. The RMSD maps of the selected compounds complexed with the SRA domain of E3 ubiquitin-protein ligase UHRF1(PDB: 3BI7) measure the average change in the positions of the atoms of the protein and ligand inside. For compound Chicoric acid, the RMSD of the protein and Chicoric acid laid over each other, indicating increased stability of the UHRF1-Chicoric acid complex (Figure 4A). Additionally, the fluctuation seen for both over the 100 ns was within the range. A similar RMSD pattern was observed for Nystose and UHRF1 complex, despite the sudden, non-significant fluctuation of Nystose at around 80 ns (Figure 5A).

The secondary structure of UHRF1(PDB: 3BI7) was also evaluated throughout the simulation while complexed with each ligand. Figures 4B, Figure 5B represented the protein evaluation while complexed with Chicoric acid and Nystose. The top plot showed the distribution of the SSE (α-helices and β-sheets) throughout the protein, represented by the residue index. The middle plot monitored the overall %SSE, while the bottom plot evaluated each SSE throughout the simulation. Both plots indicated that the overall %SSE of the protein was maintained, and each SSE was stable over the simulation.

**FIGURE 6:**
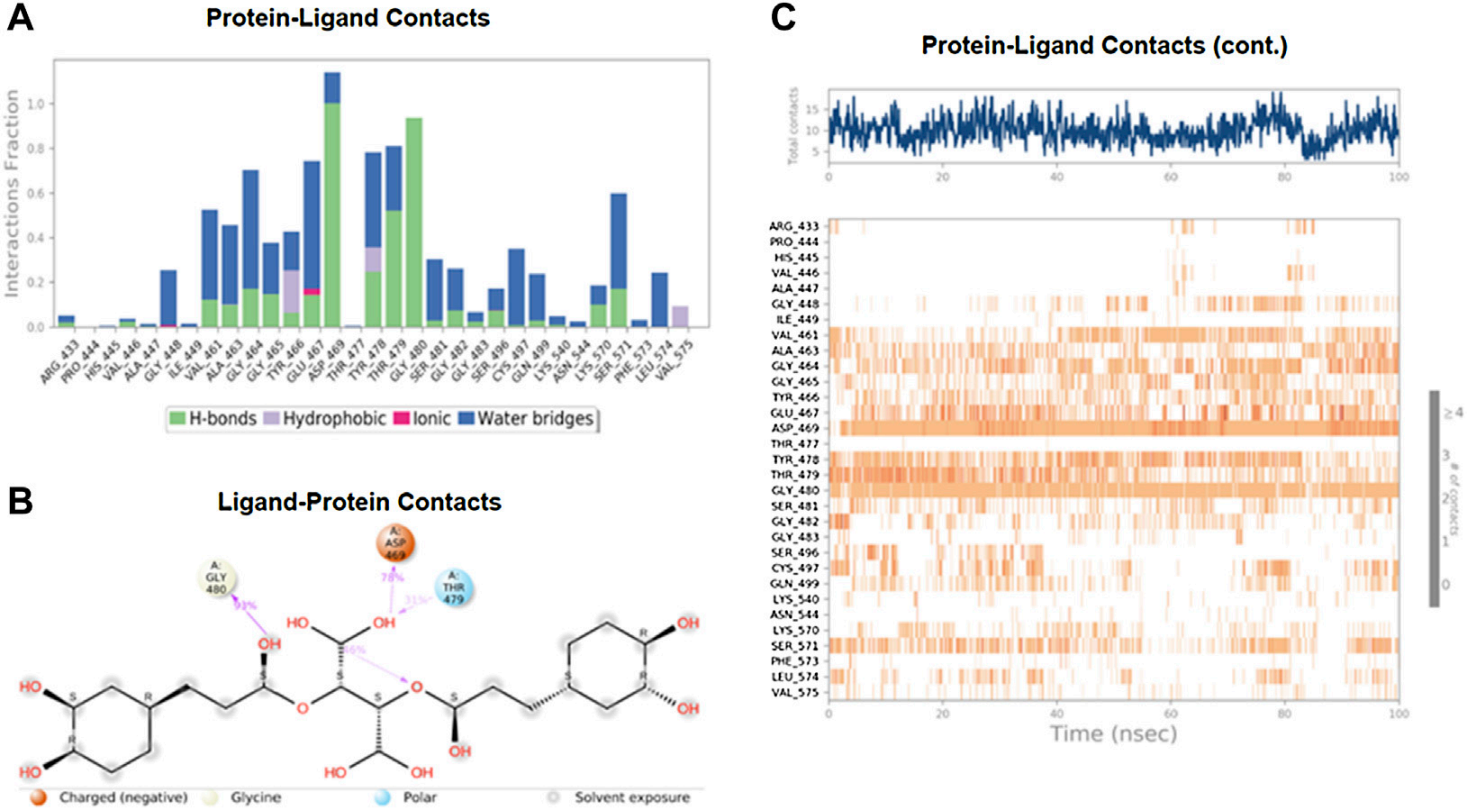
The interaction analysis of chicoric acid with UHRF1. **(A)** UHRF1 interactions with Chicoric acid throughout the simulation. The interactions between the ligand and protein were classified into hydrophobic, ionic, hydrogen bonds, and water bridges. Each classification can be further sub-grouped and noticed in the “Simulation Interactions Diagram” panel. The stacked bar charts were normalized over the trajectory’s course: for example, a value of 0.7 suggested that the specific interaction was maintained 70% of the simulation time. Values over 1.0 were possible, as some protein residue may make multiple contacts of the same subtype with the ligand. **(B)** The schematic diagram showed the detailed atomic interaction of Chicoric acid with UHRF1. Interactions occurred more than 30.0% of the simulation time in the selected trajectory (0.00 through 100.00 ns). It is possible to have interactions with >100% as some residues may have multiple interactions of a single type with the same ligand atom. **(C)** A timeline representation of UHRF1- Chicoric acid interactions is presented in **(A)**. The top panel showed the number of specific contacts that the protein made with the ligand throughout the trajectory. The bottom panel showed which residues interacted with the ligand in each trajectory frame. According to the scale of the plot, some residues made more than one specific contact with the ligand, which was represented by a darker shade of orange.

The MD study also evaluated the binding interactions of a protein-ligand complex. For the ligand Chicoric acid, the bar graph represented what type(s) of interactions the amino acid residues in the binding pocket made with the ligand and for how long the interaction was maintained throughout the simulation. The interactions were color-coded in the stacked bar graph, as indicated in Figure 6A. Asp-467 made direct H-bonding and through water bridges with Chicoric acid and had a normalized value of ∼1.2. The value > 1 represented the combined value of >1 type of interaction, indicating that these interactions were maintained for ∼120% of the simulation time. The other vital interactions were Gly-464, Glu-467, Tyr-478, Thr-479, Gly-480, and Ser-571, with value of ∼0.7, ∼0.76, ∼0.8, ∼0.82, ∼0.95, and ∼0.6, respectively. Figure 6B showed only the interactions between Chicoric acid and the protein that occurred ≥30% of the simulation time. Figure 6C displayed the specific interactions between ligand Chicoric acid and the protein (top plot). At the same time, the bottom panel demonstrated the protein residues that interacted with the ligand at each time point/trajectory. If a residue makes more than one specific interaction with the ligand, it appears as darker orange color in the plot. As mentioned earlier, Asp-469 made >1 interaction with the ligand, represented by the dark orange color in the plot throughout the trajectory.

**FIGURE 7:**
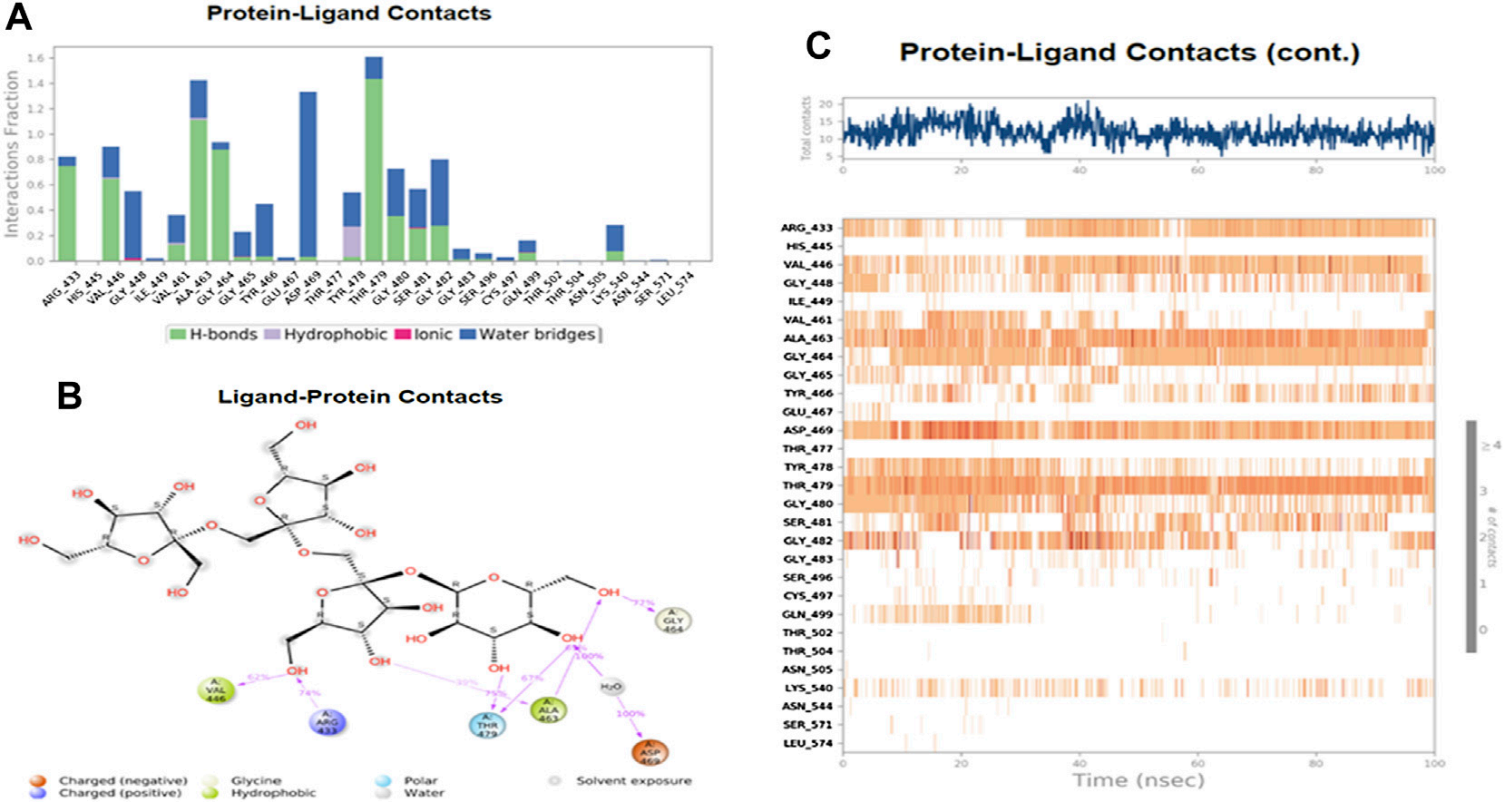
The interaction analysis of nytose with UHRF1. **(A)** UHRF1 interactions with Nystose throughout the simulation. **(B)** The schematic diagram showed the detailed atomic interaction of Nystose with UHRF1. **(C)** A timeline representation of UHRF1- Nystose interactions is presented in **(A)**.

Figure 7 shows the amino acid residues of the protein binding pocket that interacted with Nystose. Val-446, Ala-463, and Thr-479 made direct H-bonding, and through water, bridges with Nystose had a normalized value of ∼0.94, ∼1.50, and ∼1.63. The other vital interactions were with Arg-433, Gly-464, Asp-469, Gly-480, and Gly-482, with values of ∼0.82, ∼0.96, ∼1.38, ∼0.77 ∼0.82, respectively.

### Analysis of *In Silico* Toxicity

Our *in-silico* toxicity results for chicoric acid nystose showed that the compounds belong to the toxicity class 5 with LD50 of 5000 mg/kg and 3000 mg/kg respectively. The various toxicity model reports that include hepatotoxicity, carcinogenicity, stress response pathways, etc, were depicted and represented in Table 2.

**TABLE 2:** Toxicity analysis of chicoric acid with different parameters such as, organ toxicity, toxicity endpoints, and signaling and response pathways.

Classification	Target	Chicoric acid	Nystose
Oral toxicity	LD50 (mg/kg)	5000	3000
Organ toxicity	Hepatotoxicity	Inactive	Inactive
Toxicity end points	Carcinogenicity	Inactive	Inactive
	Immunotoxicity	Active	Inactive
	Mutagenicity	Inactive	Inactive
	Cytotoxicity	Inactive	Inactive
Tox21-Nuclear receptor signalling pathways	Aryl hydrocarbon Receptor (AhR)	Inactive	Inactive
Tox21-Nuclear receptor signalling pathways	Androgen Receptor (AR)	Inactive	Inactive
Tox21-Nuclear receptor signalling pathways	Androgen Receptor Ligand Binding Domain (AR-LBD)	Inactive	Inactive
Tox21-Nuclear receptor signalling pathways	Aromatase	Inactive	Inactive
Tox21-Nuclear receptor signalling pathways	Estrogen Receptor Alpha (ER)	Inactive	Inactive
Tox21-Nuclear receptor signalling pathways	Estrogen Receptor Ligand Binding Domain (ER-LBD)	Inactive	Inactive
Tox21-Nuclear receptor signalling pathways	Peroxisome Proliferator Activated Receptor Gamma (PPAR-Gamma)	Inactive	Inactive
Tox21-Stress response pathways	Nuclear factor (erythroid-derived 2)-like 2/antioxidant responsive element (nrf2/ARE)	Inactive	Inactive
Tox21-Stress response pathways	Heat shock factor response element (HSE)	Inactive	Inactive
Tox21-Stress response pathways	Mitochondrial Membrane Potential (MMP)	Inactive	Inactive
Tox21-Stress response pathways	Phosphoprotein (Tumor Supressor) p53	Inactive	Inactive
Tox21-Stress response pathways	ATPase family AAA domain-containing protein 5 (ATAD5)	Inactive	Inactive

### Global Methylation Level Reduced by Chicoric Acid

**FIGURE 8:**
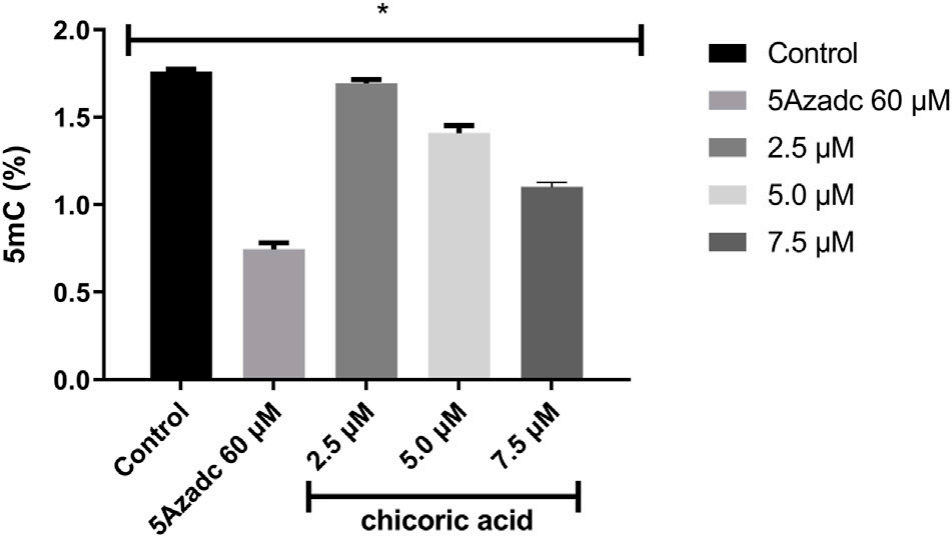
The global genomic methylation level study in chicoric acid treated HCT116 cell line. The percentage of global methylation (5mC) of HCT116 cell line treating with chicoric acid. The concentration of 7.5 µm showed the lowest percentage of methylation (5mC) level. 5Azadc; 5Azacytidine.

The level of global methylation (5mC) in HCT116 cell line was determined by using the computationally identified chicoric acid. Based on a previous study (Sun et al., 2019), the methylation level was calculated at three different doses of chicoric acid, such as 2.5, 5 and 7.5 µM. Moreover, 5Azacytidine (as a positive control of DNMTi) treatment was also performed for assessing the %5 mC level (Nur et al., 2022). The data illustrated that a 2.5 µM dose had a very low effect on the 5 mC level reduction relative to the control sample. Moreover, the methylation (5mC) level was moderately decreased by 5 µM chicoric acid treatment. However, 7.5 µM chicoric acid treatment significantly reduced the 5 mC level by around 0.6% compared to the control (Figure 8).

## Discussion

DNMTs have profound epigenetic effects on various tumorigenic and non-tumorigenic cells (Robertson, 2001). Besides a coordinating protein like UHRF1 interact with DNMTs through SAR domain. Previously, it has been shown that targeting the SRA domain of UHRF1 with various natural compounds provide a promising strategy for chemotherapeutic purpose in cancer cell lines (Patnaik et al., 2018). However, the study of targeting the SRA domain of UHRF1 by chicoric acid has been overlooked previously. Hence, in our present study, we aimed to investigate this gap. First, we retrieved the natural compounds library followed by virtual screening through molecular docking simulation. In molecular docking simulation, we virtually screened 709 natural organic compounds against the SRA domain of UHRF1. After molecular docking simulations, we selected the top two compounds based on the docking score which include chicoric acid and nystose. The molecular docking simulation result of chicoric acid showed that the chicoric acid interacted with 14 amino acids of SRA domain UHRF1 whereas nystose interacted with 16 residues including Asp469 residue. Asp469 of SRA domain is studied as an active residue that recognizes methylcytosine (Patnaik, 2020). The study showed that chicoric acid formed significant interactions with Asp469 while the interaction with nystose is very low. Furthermore, the study utilized molecular dynamics simulation to analyze the protein-ligand complex stability (Islam et al., 2022). Also, MD simulation calculates the RMSD values to confirm the interaction stability and rigidity of the compounds with the target protein (Liu et al., 2017). The RMSD value of the SRA domain-chicoric acid complex showed more stable considerably. Besides the interaction mapping also chicoric acid confirmed more stable and durable H-bonding with Asp469 residue throughout 100ns simulation. Moreover, we also investigated the *in-silico* toxicity test that showed both chicoric acid and nystose meet all the safety parameters and belong to toxicity class 5. However, chicoric acid has been characterized as a more selective candidate with a higher LD50 value of 5000 mg/kg. Previously, chemoinformatics study of chicoric acid has been studied targeting various proteins (Healy et al., 2009; Baskaran et al., 2012; Li et al., 2021). Till now no chemoinformatics study of chicoric acid-targeting SRA domain of UHRF1 has been elucidated.

Additionally, the global methylation level (5mC%) was measured to validate our *in-silico* results (De Oliveira et al., 2020). We tested the chicoric acid on the HCT116 cell line at 2.5, 5 and 7.5 µM doses. From the treated sample we have extracted the genomic DNA to measure the global methylation level of the genome (5mC%). Our results showed that at 7.5 µM treatment chicoric acid reduced the highest level of 5mC. No previous study showed the effect of chicoric acid on global methylation levels.

## Conclusion

DNA methylation is necessary to control the mammalian genome expression and stability. However, aberrant DNA methylation leads to carcinogenesis. UHRF1 is found as a crucial target for facilitating uncontrolled methylation. Particularly, SRA domain of UHRF1 is highly responsive to incorporating other epigenetic writers including DNMT1. Therefore, our study utilized diverse *in-silico* approaches and virtually screened 709 natural compounds that showed chicoric acid and nytose as prominent interactors with or without the SRA domain of UHRF1 protein and finally identified chicoric acid as a promising drug candidate against the SRA domain. Finally, chicoric acid justified the epi-drug-like effect of chicoric acid on HCT116 cancer cell line by measuring the global methylation level (5mC%). Chicoric acid was substantially effective in reducing DNA methylation levels suggesting that chicoric acid may become a new epigenetic inhibitory drug for chemotherapeutic purposes in cancer treatment.

## Data Availability

The raw data supporting the conclusion of this article will be made available by the authors, without undue reservation.
